# Therapeutic Strategies for Attenuation of Retinal Ganglion Cell Injury in Optic Neuropathies: Concepts in Translational Research and Therapeutic Implications

**DOI:** 10.1155/2019/8397521

**Published:** 2019-11-11

**Authors:** Lin Fu, Sum Sum Kwok, Yau Kei Chan, Jimmy Shiu Ming Lai, Weihua Pan, Li Nie, Kendrick Co Shih

**Affiliations:** ^1^Affiliated Eye Hospital, School of Ophthalmology and Optometry, Wenzhou Medical University, Zhejiang, Wenzhou, China; ^2^Department of Ophthalmology, Li Ka Shing Faculty of Medicine, University of Hong Kong, Hong Kong, China

## Abstract

Retinal ganglion cell (RGC) death is the central and irreversible endpoint of optic neuropathies. Current management of optic neuropathies and glaucoma focuses on intraocular pressure-lowering treatment which is insufficient. As such, patients are effectively condemned to irreversible visual impairment. This review summarizes experimental treatments targeting RGCs over the last decade. In particular, we examine the various treatment modalities and determine their viability and limitations in translation to clinical practice. Experimental RGC treatment can be divided into (1) cell replacement therapy, (2) neuroprotection, and (3) gene therapy. For cell replacement therapy, difficulties remain in successfully integrating transplanted RGCs from various sources into the complex neural network of the human retina. However, there is significant potential for achieving full visual restoration with this technique. Neuroprotective strategies, in the form of pharmacological agents, nutritional supplementation, and neurotrophic factors, are viable strategies with encouraging results from preliminary noncomparative interventional case series. It is important to note, however, that most published studies are focused on glaucoma, with few treating optic neuropathies of other etiologies. Gene therapy, through the use of viral vectors, has shown promising results in clinical trials, particularly for diseases with specific genetic mutations like Leber's hereditary optic neuropathy. This treatment technique can be further extended to nonhereditary diseases, through transfer of genes promoting cell survival and neuroprotection. Crucially though, for gene therapy, teratogenicity remains a significant issue in translation to clinical practice.

## 1. Introduction

Retinal ganglion cell (RGC) death is the final common pathway in a number of optic neuropathies of various causes, including glaucoma, demyelinating optic neuritis, ischemic optic neuropathy, and hereditary optic neuropathy. For sufferers of optic neuropathies, RGC death remains irreversible with existing therapeutic strategies [1]. Among optic neuropathies, glaucoma is the most prevalent, being the second leading cause of blindness worldwide [2]. Currently, only therapeutic strategies for lowering intraocular pressure (IOP), including eyedrops, laser, and drainage surgeries, are clinically available for slowing glaucoma disease progression. Furthermore, despite the use of IOP-lowering treatment, a significant number of glaucoma patients under management still progress to irreversible blindness [3]. For ischemic and traumatic optic neuropathies, there remains a lack of viable treatment strategies for sufferers. While idebenone is approved in Europe for slowing disease progression and improving visual outcomes in patients with Leber's hereditary optic neuropathy (LHON), the condition remains incurable.

In the recent decades, there have been a number of proposed IOP-independent therapeutic strategies for RGC-related optic neuropathies. Overall, the objectives of these treatments are to ameliorate optic neuropathies by providing a nourishing environment for damaged RGCs and/or to replace damaged or dead RGCs with healthy new ones. While a number of therapeutic strategies have demonstrated promising results in vitro and in animal models of optic neuropathy, as well as in noncomparative interventional case series, there remains significant issues affecting their translation to clinical practice. In this paper, we aim to summarize and critically appraise translational research in this field and discuss the potential implications of such treatments to clinical practice.

## 2. Methodology

Therapeutic strategies were classified into those belonging to (1) cell therapies, (2) noncellular neuroprotective therapy, and (3) gene delivery-based neuroprotective therapy. The treatments were further divided into subheadings within each of the three categories.

### 2.1. Cell Therapies

There are three major types of cell replacement therapy, including human Müller glia cells- (hMGCs-) derived RGCs, human pluripotent stem cell- (PSC-) derived RGCs, and mesenchymal stem cell (MSC) transplantation. The former two directly deliver RGCs to areas of cell loss and the latter one transplants a neurotrophic environment to the area of injury in support of the injured RGCs. Cell replacement can resolve two major problems. Firstly, it offers a nourishing environment for damaged RGCs in order to retard or prevent secondary degeneration and subsequent visual impairment [1, 2]. Secondly, albeit more ambitiously, the aim of cell replacement therapy is to replace damaged cells with healthy functioning ones.

#### 2.1.1. Cell Replacement

Retina Müller glia are cells native to the retina and, with an appropriate microenvironment, can be manipulated to differentiate into RGCs in vitro [3, 4]. In 1998, RGC precursor cells were successfully derived from hMGCs by in vitro inhibition of cellular Notch activity [5]. In experimental RGC-depletion models, transplantation of hMGC-derived RGCs resulted in higher negative scotopic threshold responses on ERG in mice and cats with damaged optic nerves compared to untreated controls, demonstrating significantly improved RGC function after transplantation [4, 6]. However, the exact mechanism of hMGC reprogramming to RGC precursor cells remains to be understood. It has been suggested that cytokines, including tumor necrosis factor-*α* (TNF-*α*) and growth factors, such as heparin-binding epidermal growth factor-like growth factor a (Hbefga), are secreted in response to RGC injury and are necessary in the microenvironment for MGC reprogramming [7, 8]. For this to be a viable form of treatment for patients with optic neuropathy, the holy grail for research in the field would be endogenous MGC-reprogramming into RGCs. Although in certain animals, such as zebrafish, Müller glia can be directly reprogrammed into progenitor cells in response to retinal injury and therefore repair damaged tissue by sprouting de novo neurons in vivo, mammals do not have this regenerative ability. Thus, reprogramming of hMGCs must be conducted ex vivo after extraction [9, 10]. There are several significant barriers towards translating this technology to clinical practice. Firstly, it is difficult to maintain Müller glia cells in their undifferentiated state in vitro, owing to their propensity to rapidly differentiate into cells of multiple lineages. Secondly, extraction of hMGCs, while avoiding damage to the donor retina, would be technically challenging with current resources. Therefore, this mode of cell replacement therapy remains a nonviable option for clinical practice.

Pluripotent stem cells possess the ability to differentiate into cells of all lineages and can be easily maintained in vitro. Therefore, PSCs have the potential to provide a theoretically unlimited source of RGCs. Langer et al. were the first to successfully identify and characterize the RGC subtype from the human pluripotent stem cell (hPSC) [11]. In the past, the main source of PSCs was embryonic stem cells (ESCs), derived through destruction of embryos. This is a controversial international issue, and many governing bodies have either banned the research altogether or placed restrictions on what may be done with embryos and ESCs. In 2006, a pioneering study from Takahashi and Yamanaka successfully demonstrated a technique to reprogram adult somatic cells into pluripotent stem cells [12]. The introduction of the technique for induced pluripotent stem cells (iPSC) was a landmark achievement that opened new doors for autologous cell replacement therapy, requiring the harvesting of only donor somatic cells, thereby avoiding significant ethical concerns with ESC research.

In visual sciences, there are three main techniques to derive RGCs from PSCs. The first technique is direct differentiation from PSC to RGC in vitro through chemical induction. However, this technique still has a relatively inefficient yield of approximately 30% [13]. The second technique is through genetic manipulation. Overexpression of genes Pax6 and Atoh/Math5 have been shown to trigger RGC differentiation from mouse ESCs and iPSCs in vitro [14–16]. A third and promising technique is differentiation through the use of a mechanical scaffold. This involves three-dimensional (3D) cell culturing techniques to promote self-differentiation and -organization into an optic cup-like multilayer structure [17, 18]. This technique has the added theoretical advantage of allowing for subsequent transplantation of entire organized retinal layers, rather than RGCs alone, and may avoid the complex culturing conditions for RGC isolation in vitro.

One important challenge of the RGC transplantation is to successfully integrate the RGCs into the complex neurological network of the host retina. Precise connections of transplanted RGC axons with recipient presynaptic amacrine, bipolar cells, and postsynaptic neurons in central nervous system are critical for success. Another formidable challenge is rejection of engrafted donor RGCs. The use of autologous transplantation from recipient somatic cell-derived iPSCs may solve this issue. However, currently, techniques for iPSC generation and differentiation are extremely time consuming and costly, preventing it from being a viable solution for clinical therapy. Although obstacles remain, if successful, RGC transplantation offers the best hope for full restoration of visual function. In addition, with all stem cell therapies, there is a serious concern of possible tumorigenesis since these pluripotent stem cells share molecular phenotypes with cancer cells [19]. Though the use of induced pluripotent stem cells is far less ethically controversial, iPSCs have a higher propensity to tumorigenesis due to epigenetic differences [19]. Regarding safety, hESC-RPE cells have been successfully transplanted into the subretinal space of rats for up to 200 days without evidence of tumorigenesis [20], . However, similar experiments have not yet been performed for RGCs derived from iPSCs or ESC.

#### 2.1.2. Cell Therapy-Based Neuroprotection

While mesenchymal stem cells do not replace damaged cells, they have been readily shown to have potent neuroprotective effects after transplantation. This effect has been demonstrated in animal models of RGC degenerative diseases [21, 22]. The underlying molecular mechanisms are related to the secretion of neurotrophic factors, including ciliary neurotrophic factor (CNTF), glia cell-derived neurotrophic factor (GDNF), and brain-derived neurotrophic factor (BDNF), by transplanted MSCs [23–28]. Adipose tissue-derived MSCs (AT-MSCs) and bone marrow-derived MSCs (BM-MSCs) are the two major sources of mesenchymal stem cells [29]. AT-MSCs have the additional advantages over BM-MSCs in being easier and less painful to be harvested, allowing for a higher yield and better ex vivo expansion [30–33]. Like all cell replacement therapy techniques, engraftment and integration of transplanted cells to the host retina is a significant problem for MSCs. However, as MSC therapy has a greater possibility of autologous transplantation than other stem cell therapies, it avoids the problem of cell rejection and ethical issues associated with embryo use. Nevertheless, four types of delivery techniques have been reported in the literature to be safe and effective for MSC transplantation, including intravitreal injection, intravenous injection, subretinal injection, and transfer of MSCs on a thin epiretinal membrane [22, 34, 35]. The relative differences in efficacy between techniques require further research for a better understanding. For example, if MSCs are delivered intravitreally, the internal limiting membrane (ILM) forms a natural barrier to retinal penetration [36]. As such, a vitrectomy procedure with ILM peeling may improve the migration and penetration of intravitreally injected MSCs to the target location.

### 2.2. Noncellular Neuroprotective Therapies

While RGC death is the central process of all optic neuropathies, the underlying etiologies can vary significantly, including ischemia-reperfusion injury, mechanical damage, inflammation, and degeneration. Therefore, a number of different neuroprotective strategies have been designed to specifically target these etiologies, including vascular regulation through inhibition of nitric oxide synthase [37, 38], depression of oxidative stress [39, 40], suppression of glutamate-induced excitotoxicity [41, 42], and modulation of glial cells activity [43]. For these neuroprotective agents, we will divide them into pharmacological agents, nutritional supplements, and neurotrophic factors.

#### 2.2.1. Pharmacological Agents

One proposed pathological mechanism of RGC death in glaucoma is excessive glutamate release. In glaucoma patients, studies have shown that intravitreal and retinal levels of glutamate and glutamine are reduced compared with controls [44, 45], while the levels of the glutamate transporter EAAT-1 are elevated. Furthermore, increased levels of glutamate has been shown to induce RGC death in vitro and in animal models through overstimulation of N-methyl-D-aspartate (NMDA) receptors [46–49], thus providing a therapeutic target for inhibition. Memantine is an uncompetitive NMDA antagonist approved by the United States Food and Drug Administration (USFDA) in treatment of Alzheimer's disease. It has been also studied in animal models of glaucoma with promising neuroprotective effects [50–53]. However, a four-year phase-3 clinical trial, investigating the therapeutic effects of daily oral intake of memantine, failed to meet the primary endpoint in subjects with open angle glaucoma at risk to disease progression [53]. However, there were several potential factors affecting drug efficacy, including baseline glaucoma severity, the study duration, the dosage of memantine used, and the route of drug administration. Further studies with earlier intervention, a less heterogenous sample, a shortened study period, and alternative dosages and routes for drug administration are warranted.

Brimonidine is a highly effective topical IOP-lowering eyedrop. It is an *α*2 adrenergic receptor agonist that increases aqueous outflow and decreases aqueous production. Furthermore, it has been shown to slow visual field progression more effectively for low-tension glaucoma subjects compared with 0.5% timolol eyedrops [54]. Therefore, it has been proposed that in addition to its potent IOP lowering effects, brimonidine may have IOP-independent neuroprotective effects as well. There are a number of proposed mechanisms from published research to account for this theoretical neuroprotective effect, including elevation of local neurotrophic factors [55], reduction of cyclic adenosine monophosphate (cAMP) [56], neuromodulation of NMDA receptors [57], and regulation of amyloid beta pathways [57]. Based on this evidence, robust randomized placebo-controlled trials with large sample sizes will be needed to confirm brimonidine's purported neuroprotective effects in clinical practice.

However, it is worth noting that these medications are not without side effects. Brimonidine's side effects include allergic conjunctivitis, blepharitis, and conjunctival hyperemia [58] with reports of severe corneal side effects such as corneal neovascularization and corneal opacification [58]. Memantine's common side effects include fatigue, pain, hypertension, dizziness, vomiting, headache, vomiting, and hallucinations [59]. Despite their respective side effects, the pharmacological options available are generally well-tolerated and serve as a feasible therapeutic option.

#### 2.2.2. Nutraceutical Supplementation

Nutritional supplementation for health and disease has been practiced in the East for generations. In recent decades, this practice has become increasingly popular in the West as part of the alternative medicine movement. Unlike pharmacological agents, there is less regulatory control over the sales and marketing of nutritional supplements, making them widely available for purchase in supermarkets and convenience stores worldwide. However, this laxity of regulations also means that much of their marketed effects are not based on evidence from well-designed clinical trials.

Ginkgo biloba extract (GBE) is a widely used nutritional supplement for the treatment of cognitive disorders like Alzheimer's dementia and has been studied in experimental models of ischemic stroke [60–62]. EGb761 is a standardized form of GBE, after removing the toxic ginkgolic acid, and contains the active component of flavonoids and terpenoids. It has been investigated as a treatment for glaucoma [63]. In preliminary clinical trials, GBE intake has been shown to slow visual field progression in subjects with normal tension glaucoma compared to controls without significant changes in IOP [64]. The involved mechanisms are purported to be ocular blood flow homeostasis [64], inhibition of glutamate release [65], and suppression of lipid peroxidation [66]. However, GBE has documented side effects such as stomach pain, headache, dizziness, constipation, palpitations, and allergic skin reactions with more serious side effects including possible drug interaction with anticoagulants or antiplatelets which may lead to increased risk of hemorrhage [67].

Coenzyme Q10 (CoQ10) is an electron carrier for mitochondrial oxidative phosphorylation. It serves as a lipid-soluble antioxidant to protect damaged mitochondrial DNA, proteins, and lipids. CoQ10 has been shown to be neuroprotective in neurogenerative disorders including Parkinson's disease, Huntington's disease, and Leber's hereditary optic neuropathy [68]. It has been further shown to improve RGC survival in experimental animal models of glaucoma and optic nerve injury [69–71]. The underlying mechanisms are inhibition of glutamate excitotoxicity, suppression of oxidative stress, and enhancement of bioenergetic function in the optic nerve head astrocytes [69, 72]. In a published clinical trial, topical application of CoQ10 in conjunction with vitamin E improved the retinal electrophysical function of open angle glaucoma patients [73]. Furthermore, in pseudoexfoliative glaucoma patients, the addition of topical CoQ10 and vitamin E reduced aqueous levels of superoxide dismutase (SOD) [74]. Whether this translates to clinical and visual benefits in glaucoma patients remains to be seen. Coenzyme Q10 supplements are extremely well-tolerated with reports of minor gastrointestinal discomfort like stomach pain, nausea, vomiting, and diarrhea [75].

Citicoline is an intermediate compound of phospholipids. In 1989, Pecori Giraldi et al. first reported in a noncomparative interventional case series that intramuscular injection of citicoline improved the visual function up to 75% in glaucomatous eyes as demonstrated by perimetric methods [76]. More recently, electrophysiological studies, using pattern electroretinogram (PERG) and visual evoked potential (VEP) recordings, have shown that intramuscular injection, oral intake, and topical application of citicoline enhanced bioelectrical responses of retina and visual cortex [77–79]. In animal models, citicoline was shown to protect RGCs in mitochondria-dependent and glutamate-mediated cell death mechanisms [80, 81]. Well-designed randomized controlled trials will be required to determine the clinical efficacy of citicoline. Citicoline is also well-tolerated with reports of minor digestive tolerance after its use [82].

Crocin, which is the pharmacologically active substance in saffron, shown to have antioxidizing effects, has been shown to reduce RGC death [83]. Intraperitoneal crocin injection was given after retinal injury in rats and showed that intraperitoneal crocin injections significantly slowed the reduction in retinal thickness with significant reduction in caspase-3 and p-ERK via immunofluorescence and western blot compared with the control group [83].

#### 2.2.3. Neurotrophic Factors

Neurotrophic factors are reported to support the growth, repair, and survival of neurons. The main factors include nerve growth factor (NGF), BDNF, CNTF, GDNF, and neurotrophin-4/5 (NT-4/5). They have been shown in published studies to have potential therapeutic effects in neurodegenerative diseases of the central nerve system (CNS). These include Huntington's disease, Alzheimer's disease, Parkinson's disease, amyotrophic lateral sclerosis (ALS), and also glaucoma [84–86]. Either through exogenous application of these neurotrophic factors or through induction of endogenous expression via viral vectors or through electrical stimulation has been shown to improve survival of RGCs in experimental models [87, 88]. Neurotrophic factors primarily interact with neurons through two different receptors, with potentially opposite effects. Through interaction with tyrosine-receptor kinases (Trk) family, they can promote cell survival, and through interaction with neurotrophin receptor p75 (p75 NTR), they can promote cell apoptosis [89].

NGF treatment has been shown to significantly improve RGC survival in an animal model of ocular hypertensive optic nerve injury [90]. Furthermore, in a small noncomparative therapeutic case series for human subjects with advanced glaucoma, ocular treatment with NGF improved optic nerve function in terms of visual field, visual acuity, and contrast sensitivity [91]. Thus, NGF is a promising neurotrophic factor that deserves further investigation through robust randomized controlled trials.

BDNF promotes RGC survival by binding to the cells' TrkB receptor. In turn, this stimulates the neuronal survival-related signaling pathway and phosphatidylinositol 3-kinasestat (PI3K/Akt), as well as extracellular signal-regulated kinase (ERK) [92, 93]. In patients with glaucoma, optic nerve, tears, and serum BDNF levels are significantly reduced compared with controls [94–96]. The localized deficiency of BDNF in glaucoma patients has been shown to be potentially a result of axonal transportation blockade as a result of optic nerve dysfunction. Therefore, serum and tear levels of BDNF may serve as a promising biomarker for glaucomatous progression. Regarding therapeutic trials with BDNF, as the neurotrophic factor cannot cross the blood brain barrier, there have not been successful reported studies showing therapeutic effects with BDNF treatment.

CNTF is natively expressed by retinal Müller glial cells. A published study demonstrated lower levels of CTNF in the tears, aqueous humor, and serum of patients with primary open angle glaucoma (POAG) compared with controls [97]. In vitro, the addition of CNTF increases retinal pigment epithelial cell secretion of levels of neurotrophin 3 (NT3) and reduces production of vascular endothelial growth factor (VEGF), transforming growth factor b2 (TGFb2), and interleukin-8 (IL-8) [98]. The purported downstream effects of CNTF are mediated through the signaling pathways of mitogen-activated protein kinase (MAPK)/ERK, Janus kinase/signal transducer and activator of transcription (JAK/STAT), and (PI3K)/Akt [89]. A published animal study investigating the therapeutic efficacy of systemic administration CNTF showed no detectable neuroprotective effects [99]; thus, an encapsulated cell implant containing genetically modified CNTF-secreting cell line is being investigated as a more effective method of drug delivery [100, 101].

In addition, vascular endothelial growth factor A (VEGF-A) has also shown neurotropic and neuroprotective effects, specifically VEGF_121_ and VEGF_165_ isoforms [102, 103]. Intravitreal VEGF-A165b injection has been shown to protect RGC from ischemia-induced death after ischemic-reperfusion injury in rats and is believed to be mediated via VEGFR2 and MEK1/2 activation. VEGF-A165b additionally protects hippocampal, cortical, and cerebellar granule neurons [104]. Previous studies have demonstrated that the proangiogenic VEGF-A165a isoform also has neuroprotective effects for hippocampal, dorsal root ganglia, and retinal neurons, but its use clinically is limited by the proangiogenic properties [104].

Moreover, growth hormones have also been shown to be neuroprotective in in vitro culture of chick embryo retinal ganglion cells [105]. Growth hormone treatment has shown to increase Akt phosphorylation which has antiapoptotic effects and serves as caspase and calpain inhibitors [105]. The prospect of intravitreal growth hormone injections as a possible future therapy to prevent RGC injury is promising, but the possible side effects are still poorly understood.

Studies on neurotrophic factors display the strong potential of their neuroprotection, although their clinical use is limited by the effective delivery to the target area. An alternative to this is induction of local secretion of neurotrophic factors through external treatments, including electrical stimulation.

### 2.3. Gene Delivery-Based Neuroprotective Therapies

Since RGCs do not regenerate after injury, gene therapy aimed at delivering normal genes to the affected eyes has the potential to rescue vision in RGC-related diseases. One famous case is the clinical trial conducted on patients with LHON. The adeno-associated virus type 2 (AAV2) carrying the NADH dehydrogenase subunit 4 (ND4) is used to treat the patients with ND4 mutation. These clinical trials showed encouraging preliminary results with improvement of visual function [106, 107]. Additional applications of gene therapy in the RGC protection are encoding neurotrophic factors like BDNF and CNTF to the experimental models of RGC injury [108, 109]. BDNF gene therapy has been promising but has been limited due to BDNF receptor downregulation which has been partially attributed to tropomyosin-related receptor kinase-B (TrkB) downregulation [110]. TrkB downregulation has been reported in glaucoma which potentially limits the effect of sustained or repeated BDNF delivery [110]. AAV2 vectors carrying the TrkB-2A-mBDNF were injected intravitreally into mice which were then subjected to optic nerve crush (ONC) and raised intraocular pressure 21 days after treatment [110] and showed stable expression of both transgenes with significant RGC protection compared to the null vector group while TrkB alone was insufficient in significantly improving RGC survival [110].

Sarzi et al. assessed the effect of OPA1 gene therapy by intravitreal AAV2/2-pCMV-HsOPA1 injection using a cytomegalovirus (CMV) promoter into mice with dominant optic atrophy which significantly reduced retinal ganglion cell death [111]. Analysis of the scotopic threshold responses (STRs) which assess the activity of inner retina neurons showed significant increase in the negative STR (nSTR) latencies of untreated mice compared with controls, but nSTR latency values after OVA1 gene reduced dramatically comparable to the control level signifying improvement of retinal ganglion cell activity [111].

Moreover, the delivery of antiapoptotic genes by intravitreal injection of viral vectors have displayed protective effect in RGCs [112, 113] which is important as RGC death is mediated by apoptosis as seen in glaucoma and experimental models [114]. Specifically, AAV-CAG vector expressing the caspase inhibitor, baculoviral IAP repeat containing protein-4 (BIRC4), significantly improved optic nerve axon survival in glaucomatous rats [115]. This provides an alternative approach to gene therapy targeting antiapoptotic pathways [114].

At the moment, gene therapy is still limited in terms of feasibility but serves as a promising future treatment in terms of not just improving RGC survival but improving function as well.

## 3. Conclusion

Over 100 different therapeutic strategies targeting RGCs were reported in in vitro studies, animal models, and preliminary clinical trials, but as of yet none of them have been successfully translated to clinical practice. For cell replacement therapy, difficulties remain in successfully integrating transplanted RGCs from various sources into the complex neural network of the human retina. However, the significant potential for achieving full visual restoration with this technique means that research in the field should continue. Neuroprotective strategies, in the form of pharmacological agents, nutritional supplementation, and neurotrophic factors, are viable strategies with encouraging results from preliminary clinical studies. However, most of these studies are focused on glaucoma, with few treating optic neuropathies of other etiologies. Gene therapy, through use of viral vectors, has shown promising results in clinical trials, particularly for diseases with specific genetic mutations like LHON. However, this technique can be further expanded to nonhereditary diseases through transfer of genes promoting cell survival and neuroprotection. However, for gene therapy, teratogenicity remains a significant issue in translation to clinical practice.

The limitations in translating promising experimental therapies to clinical practice are as follows: firstly, animal models do not fully replicate human physiology and disease. Neurodegenerative diseases like glaucoma are difficult to model in animals due their highly heterogenous nature in clinical practice. Also, receptors for potential therapeutic agents may be different in animals and in humans. Furthermore, experimental endpoints differ greatly between animal models and human clinical trials, with visual acuity, contrast sensitivity, and visual field being difficult to approximate in animals. In lieu of this, histological and electrophysiological assessments are usually carried out in animal models instead, which do not give translatable information on visual function. Moreover, encouraging findings from preliminary noncomparative therapeutic case series have not translated well to randomized controlled trials as the optimum starting point, treatment duration, therapeutic dose, and inclusion/exclusion criteria have yet to be determined for a number of promising neuroprotective agents.

Nevertheless, RGC treatment remains an exciting field of research with enormous potential for achieving visual restoration.

## Abbreviations

RGC: Retinal ganglion cell
IOP: Intraocular pressure
LHON: Leber's hereditary optic neuropathy
hMGCs: Human Müller glia cells
PSC: Pluripotent stem cell
MSC: Mesenchymal stem cell
TNF-*α*: Tumor necrosis factor-*α*
Hbefga: Heparin-binding epidermal growth factor-like growth factor a
ESCs: Embryonic stem cells
hPSC: Human pluripotent stem cell
iPSC: Induced pluripotent stem cells
3D: Three-dimensional
CNTF: Ciliary neurotrophic factor
GDNF: Glia cell-derived neurotrophic factor
BDNF: Brain-derived neurotrophic factor
AT-MSCs: Adipose tissue-derived MSCs
BM-MSCs: Bone marrow-derived MSCs
ILM: Internal limiting membrane
NMDA: N-methyl-D-aspartate
USFDA: United States Food and Drug Administration
cAMP: Cyclic adenosine monophosphate
GBE: Ginkgo biloba extract
CoQ10: Coenzyme Q10
SOD: Superoxide dismutase
PERG: Pattern electroretinogram
VEP: Visual evoked potentials
NGF: Nerve growth factor
NT-4/5: Neurotrophin-4/5
CNS: Central nervous system
ALS: Amyotrophic lateral sclerosis
Trk: Tyrosine-receptor kinases
p75 NTR: Neurotrophin receptor p75
PI3K/Akt: Phosphatidylinositol 3-kinase/protein kinase B
ERK: Extracellular signal-regulated kinase
POAG: Primary open angle glaucoma
NT3: Neurotrophin 3
VEGF: Vascular endothelial growth factor
TGFb2: Transforming growth factor b2
IL-8: Interleukin-8
MAPK: Mitogen-activated protein kinase
JAK/STAT: Janus kinase/signal transducer and activator of transcription
AAV2: Adeno-associated virus type 2
ND4: NADH dehydrogenase subunit 4.
